# Interventional Potential of Recombinant Feline Hepatocyte Growth Factor in a Mouse Model of Non-alcoholic Steatohepatitis

**DOI:** 10.3389/fendo.2018.00378

**Published:** 2018-07-23

**Authors:** Yoon Mee Yang, Masato Fukui, Zhijun Wang, Fiona Miao, Margo J. Karriker, Ekihiro Seki

**Affiliations:** ^1^Division of Digestive and Liver Diseases, Department of Medicine, Cedars-Sinai Medical Center, Los Angeles, CA, United States; ^2^Veterinary Medical Center–San Diego, University of California, San Diego, San Diego, CA, United States; ^3^Department of Biomedical Sciences, Cedars-Sinai Medical Center, Los Angeles, CA, United States; ^4^Division of Gastroenterology, Department of Medicine, School of Medicine, University of California, San Diego, La Jolla, CA, United States; ^5^Department of Medicine, David Geffen School of Medicine, University of California, Los Angeles, Los Angeles, CA, United States

**Keywords:** HGF, NAFLD, NASH, recombinant, inflammation

## Abstract

**Background and Aims:** Hepatocyte growth factor (HGF) is a multifunctional pleiotropic protein involved in tissue regeneration, protection, angiogenesis, anti-inflammatory and anti-fibrotic responses, and tumorigenesis, through binding to its receptor MET. Recombinant HGF protein has been shown to mitigate various liver disease models, such as alcohol-induced liver injury, hepatic ischemia-reperfusion injury, and fibrosis. This study aimed to investigate the anti-inflammatory, anti-fibrotic, and anti-lipogenic effects of exogenous administration of feline HGF on a non-alcoholic steatohepatitis (NASH) mouse model.

**Methods:** Wild-type C57BL/6 mice were fed a choline-deficient amino acid defined (CDAA) diet for 3 weeks to create the mouse model of NASH, which displays hepatic steatosis, inflammation, injury, and very mild fibrosis. One mg/kg of recombinant feline HGF was administered intravenously daily in the last 7 days of the total 3 weeks of CDAA diet feeding. Then, hepatic steatosis, inflammation, injury, and fibrogenic gene expression was examined.

**Results:** After 3 weeks of a CDAA diet-feeding, the vehicle-treated mice exhibited evident deposition of lipid droplets in hepatocytes, inflammatory cell infiltration, and hepatocyte ballooning along with increased serum ALT levels whereas recombinant HGF-treated mice showed reduced hepatic steatosis, inflammation, and ballooned hepatocytes with a reduction of serum ALT levels. Recombinant HGF administration promoted hepatocyte proliferation. Increased hepatic lipid accumulation was accompanied by elevated expression of lipogenesis genes *Fasn* and *Dgat1* in vehicle-treated mice. In HGF-treated mice, these genes were reduced with a decrease of lipid accumulation in the liver. Consistent with the anti-inflammatory property of HGF, augmented macrophage infiltration and upregulation of chemokines, *Cxcl1*, *Ccl2*, and *Ccl5* in the CDAA diet fed mice, were suppressed by the addition of the HGF treatment. Finally, we examined the fibrotic response. The vehicle-treated mice had mild fibrosis with upregulation of *Col1a1*, *Acta2*, *Timp1*, *Tgfb1*, and *Serpine1* expression. Recombinant HGF treatment significantly suppressed fibrogenic gene expression and collagen deposition in the liver.

**Conclusion:** Recombinant feline HGF treatment suppressed the progression of NASH in a CDAA diet feeding mouse model.This suggests that recombinant HGF protein has therapeutic potential for NASH.

## Introduction

Non-alcoholic fatty liver disease (NAFLD) is a hepatic manifestation of metabolic syndrome, which is characterized by excessive accumulation of fat content in hepatocytes. The development of NAFLD is highly associated with obesity and type 2 diabetes. Currently, 25% of adults suffer from NAFLD in the United States. NAFLD encompasses two clinicopathological entities, simple steatosis and non-alcoholic steatohepatitis (NASH). NASH is histologically characterized by hepatic steatosis along with inflammatory cell infiltration, ballooned hepatocytes, and pericellular, “chicken wire,” fibrosis (1–4). NASH-induced fibrosis may further progress to cirrhosis, and up to 13% of NASH patients with cirrhosis eventually develop hepatocellular carcinoma (HCC) (1–5). Currently, NASH-mediated cirrhosis is the second leading cause of liver transplantation, and it is predicted that this disease will be the leading cause of liver transplantation within the next 10 years (5). To date, lifestyle intervention, including weight loss and exercise, is the primary treatment for NAFLD, and there is no effective preventive or therapeutic drugs for NAFLD. Since there is a chronic shortage of liver donors, an unmet need for new and effective therapies for NAFLD is significant.

Not only in the human healthcare field, but also in veterinary medicine field, liver disease is a significant health concern in canines and felines (6). Hepatic lipidosis, which shares similar histological morphologies with human NAFLD, developed in the elderly, overweight cats that lost weight after not eating for days to weeks (6). This condition often leads to unfavorable clinical outcomes. Similar to that of human NAFLD, there is also a significant unmet need for effective therapies for fatty liver disease in animals.

Hepatocyte growth factor (HGF) is a multifunctional pleiotropic cytokine. HGF is initially produced as a biologically inactive, single-chain precursor form (7). After proteolytic cleavage, pro-HGF converts to the active form consisting of heterodimeric 69 kDa α-chain and 34 kDa β-chain (7). The active form of HGF promotes tissue regeneration, protection, wound healing, angiogenesis, tumorigenesis, and anti-inflammatory, anti-apoptotic, and anti-fibrotic responses in a wide variety of organs, through binding to its receptor MET. In the liver, HGF plays a crucial role in liver regeneration after hepatectomy or massive liver damage, protection against hepatocyte apoptosis and necrosis, and suppression of liver fibrosis progression (7). Previous studies reported that administration of recombinant HGF mitigated alcohol-induced liver damage, ischemia-reperfusion liver injury, endotoxin-induced fulminant hepatitis, and liver fibrosis (8–12). The studies using transgenic mice overexpressing *Hgf* and hepatocyte-specific *c-met* knockout mice, indicate the protective role of the HGF-MET pathway in the development of NAFLD (13, 14). Although there is a report with the application using HGF gene therapy on a rat model of NAFLD-fibrosis (15), to the best of our knowledge, the therapeutic effect of recombinant HGF protein on the development of NASH has not been reported.

The high-fat diet-induced fatty liver model does not develop liver injury, inflammation, and fibrosis in a short period of feeding. The feeding of methionine-choline-deficient diet, commonly used for NASH preclinical studies, significantly reduces their body weight. These models do not well-recapitulate the pathophysiology of human NAFLD. Notably, rodents fed a choline-deficient amino acid-defined (CDAA) diet for 3 weeks develop hepatic steatosis, inflammation, and mild liver fibrosis without reducing body weight (16, 17). Therefore, we decided to use CDAA diet feeding to develop a mouse model of NASH in this study.

The primary purpose of this study is to investigate the therapeutic effect of recombinant HGF protein on the progression of NASH. Since the amino acid sequence of feline HGF shows 97.5, 93.3, and 93.2% homology with those of canine, mouse, and human (18, 19), the second aim of this study is that to examine whether feline-derived recombinant HGF can be used for the treatment of animals with liver diseases using a mouse model of NASH.

## Materials and methods

### Animal experiments

Animal experiments were performed in accordance with National Institutes of Health recommendations outlined in the Guide for the Care and Use of Laboratory Animals. All animal experiment protocols were approved by the University of California San Diego Institutional Animal Care and Use Committee. Male C57BL/6 mice were purchased from The Jackson Laboratory (Bar Harbor, MA) and were maintained in a 12 h light/dark cycle. Mice at 8 weeks of age were subjected to feeding *ad libitum* either choline-supplemented L-amino acid-defined diet (CSAA; catalog #518754; Dyets Inc, Bethlehem, PA) or choline-deficient L-amino acid-defined diet (CDAA; catalog #518753; Dyets Inc) for 3 weeks. Vehicle or 1 mg/kg of recombinant feline HGF was intravenously injected daily during the last 7 days.

### Histologic examination

Mouse liver tissues were fixed in 10% neutral buffered formalin phosphate (Fisher Scientific, Pittsburgh, PA) and then embedded into paraffin blocks. 5-μm thick sections were cut on a microtome (Thermo Scientific, Waltham, MA). Tissues were stained with hematoxylin and eosin for the evaluation of NAFLD Activity Score (steatosis, lobular inflammation, hepatocyte ballooning) and fibrosis as described (20). Immunochemistry for proliferating cellular nuclear antigen (PCNA), F4/80 and Sirius Red staining were performed as previously reported (21, 22). In brief, liver sections were incubated with monoclonal antibody to PCNA (clone PC10; Biolegend, San Diego, CA) using the MOM kit (Vector Laboratories, Burlingame, CA). Sections were incubated with monoclonal antibody to F4/80 (clone BM8; eBioscience, San Diego, CA) for immunohistochemical analysis of F4/80 expression or incubated with a solution of saturated picric acid containing 0.1% Fast Green FCF (Sigma-Aldrich, St Louis, MO) and 0.1% Direct Red 80 (Sirius Red R3B; Sigma-Aldrich, St Louis, MO) for Sirius Red staining. For Oil Red O staining, mouse liver tissues were fixed in 4% neutral buffered formalin phosphate and then embedded into OCT compound. Frozen liver tissues were sliced into 5-μm sections and stained with Oil Red O. PCNA or F4/80 or Oil Red O-positive area was evaluated from randomly selected 10 fields of x200 magnification per slide and quantified with NIH Image J software.

### Serum alanine aminotransferase (ALT), triglyceride, and total cholesterol measurement

Blood was collected via cardiac puncture, centrifuged at 5,000 rpm for 15 min, and the serum was frozen immediately. Serum ALT levels were determined by Infinity ALT (GPT) liquid stable reagent (Thermo Scientific, Middletown, VA) according to manufacturer's protocol. Serum triglyceride and total cholesterol levels were determined by Triglycerides liquid reagent set (Pointe Scientific, Inc., Canton, MI) and Cholesterol E CHOD-DAOS method (Wako, Osaka, Japan), respectively.

### Quantitative real-time polymerase chain reaction (qPCR)

The total RNAs were extracted from snap-frozen mouse liver tissues using NucleoSpin^®;^ RNA kit (Macherey-Nagel, Düren, Germany). Reverse-transcribed with High-Capacity cDNA Reverse Transcription Kit (Applied Biosystems, Foster City, CA) and iTaq™ Universal SYBR^®;^ Green Supermix (Bio-rad, Hercules, CA) were used. The sequences of mouse PCR primers are listed in Table 1. Quantitative real-time PCR was performed using CFX96 real-time PCR system (Bio-rad, Hercules, CA).

**Table 1:** Primer sequence used for qRT-PCR.

**Gene**	**Forward**	**Reverse**
*18S*	AGTCCCTGCCCTTTGTACACA	CGATCCGAGGGCCTCACTA
*Fasn*	GTTGGCCCAGAACTCCTGTA	GTCGTCTGCCTCCAGAGC
*Dgat1*	TCACCACACACCAATTCAGG	GACGGCTACTGGGATCTGA
*Cxcl1*	TGCACCCAAACCGAAGTC	GTCAGAAGCCAGCGTTCACC
*Ccl2*	ATTGGGATCATCTTGCTGGT	CCTGCTGTTCACAGTTGCC
*Ccl5*	CCACTTCTTCTCTGGGTTGG	GTGCCCACGTCAAGGAGTAT
*Col1a1*	TAGGCCATTGTGTATGCAGC	ACATGTTCAGCTTTGTGGACC
*Acta2*	GTTCAGTGGTGCCTCTGTCA	ACTGGGACGACATGGAAAAG
*Timp1*	AGGTGGTCTCGTTGATTTCT	GTAAGGCCTGTAGCTGTGCC
*Tgfb1*	GTGGAAATCAACGGGATCAG	ACTTCCAACCCAGGTCCTTC
*Serpine1*	TTCAGCCCTTGCTTGCCTC	ACACTTTTACTCCGAAGTCGGT

### Statistical analysis

Differences between the two groups were compared using the Mann Whitney *U*-test or two-tailed unpaired student *t*-test. Differences between multiple groups were compared using one-way ANOVA. Statistical significance was assessed by using GraphPad Prism 5.01 software (GraphPad Software, Inc, La Jolla, Ca). *P* < 0.05 were considered significant.

## Results

### The therapeutic effect of recombinant HGF protein in a mouse model of NASH induced by CDAA diet feeding

**Figure 1:**
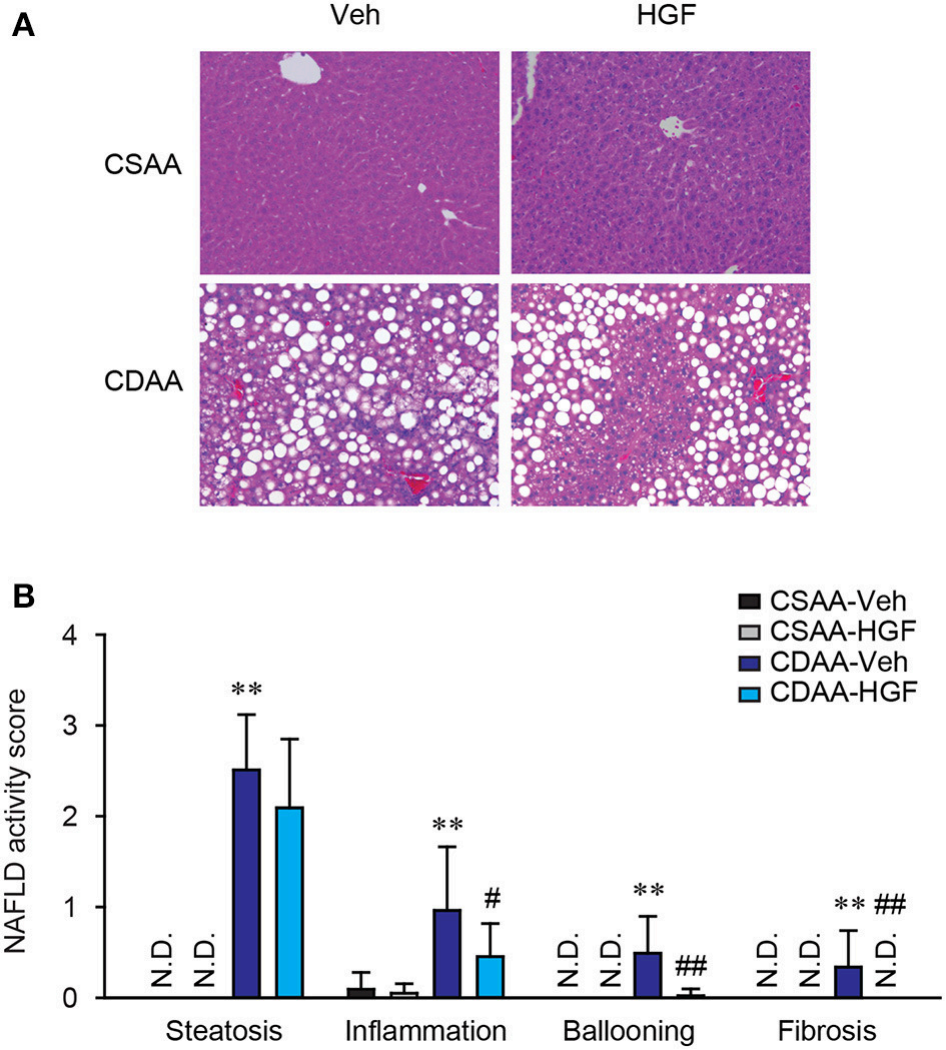
Recombinant HGF administration inhibited CDAA diet-induced NASH. Male C57BL/6 mice were fed a CSAA or CDAA diet for 3 weeks. In the last 7 days, vehicle (Veh) or 1 mg/kg of recombinant feline HGF were given intravenously daily (CSAA-Veh, *n* = 8; CSAA-HGF, *n* = 10; CDAA-Veh, *n* = 13; CDAA-HGF, *n* = 11). **(A)** H&E staining. Original magnification, x200. **(B)** NAFLD activity score. Hepatic steatosis, inflammation, hepatocyte ballooning, and fibrosis were evaluated through H&E stains. Data are presented as mean ± S.D. (**P < 0.01, significantly different from CSAA-Veh; ^#^*P* < 0.05, ^##^*P* < 0.01, significantly different from CDAA-Veh).

To investigate the therapeutic effect of recombinant HGF protein on a CDAA diet-induced mouse NASH model, wild-type C57BL/6 mice were treated with vehicle or 1 mg/kg of recombinant feline HGF protein intravenously in the last 7 days of a total of 3 weeks of CDAA diet feeding. Based on NAFLD activity scoring system, we assessed the effect of recombinant HGF on CDAA diet-induced NASH. Three weeks of CDAA diet feeding showed an evident accumulation of lipid droplets in hepatocytes, inflammatory cell infiltration, ballooned hepatocytes, and mild fibrosis in the vehicle-treated mice compared to CSAA diet feeding. The recombinant HGF treatment significantly suppressed the development of inflammation, hepatocyte ballooning, and fibrosis induced by CDAA diet feeding (Figures 1A,B). Our results indicate that recombinant HGF treatment administered on the last 7 days of CDAA diet, improved NAFLD activity score.

### Recombinant HGF administration inhibits CDAA diet-induced liver damage and increases hepatocyte proliferation

**Figure 2:**
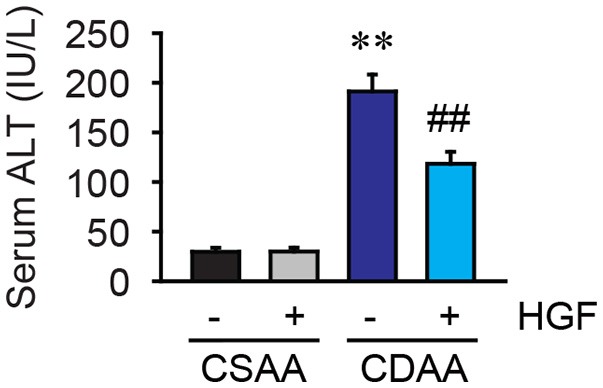
CDAA diet-mediated liver damage was suppressed by recombinant HGF treatment. Serum ALT level. Data are presented as mean ± SEM. (**P < 0.01, significantly different from CSAA-Veh; ^##^*P* < 0.01, significantly different from CDAA-Veh).

To examine the protective effect of recombinant HGF on NASH-mediated hepatocyte damage, serum ALT levels were measured. Three weeks of CDAA diet feeding dramatically elevated serum ALT levels in vehicle-treated mice, whereas increased serum ALT levels by CDAA diet feeding were significantly reduced by the administration of recombinant HGF protein (Figure 2). This result indicates that the 7 days of recombinant HGF treatment suppressed hepatocyte damage induced by the 3 weeks of CDAA diet feeding in mice.

**Figure 3:**
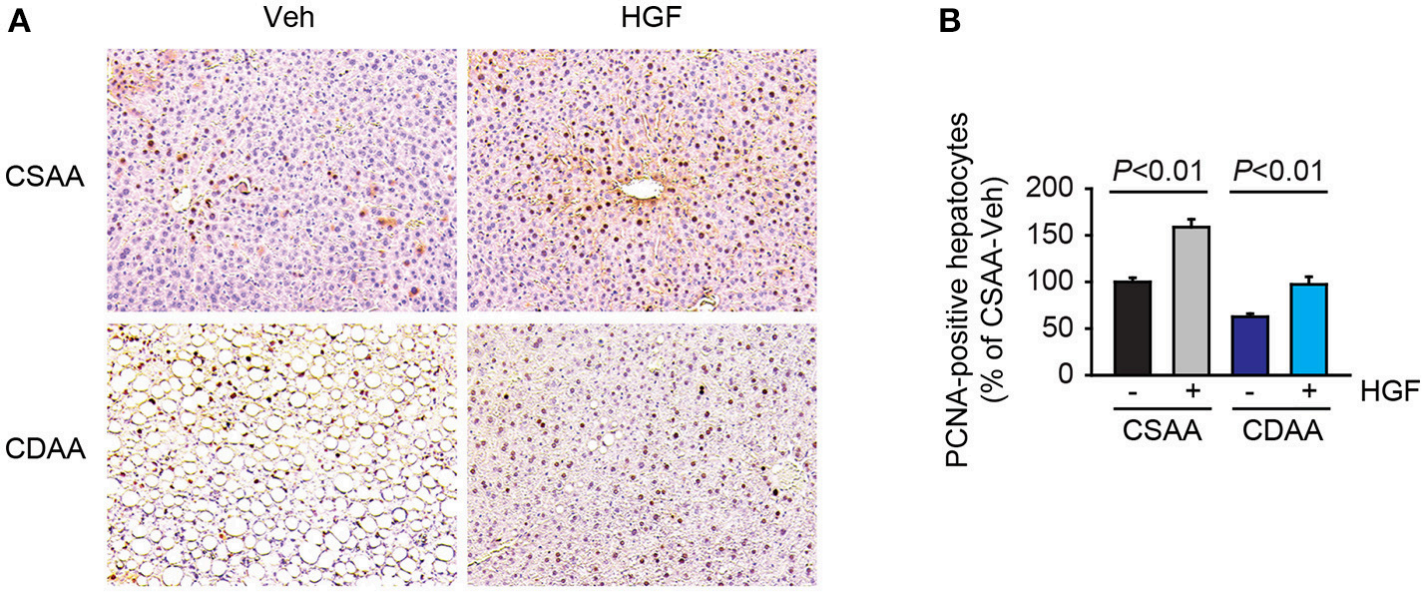
Administration of recombinant HGF promoted hepatocyte proliferation. **(A)** Representative images of PCNA staining. Original magnification, x200. **(B)** Quantification of PCNA staining. Data are presented as mean ± SEM.

Since HGF is a potent mitogen, we next evaluated the effect of exogenous HGF on hepatocyte proliferation. The increases in PCNA labeling were observed in both HGF-treated CSAA diet-fed and CDAA diet-fed mice (Figures 3A,B).

### Recombinant HGF protein has anti-lipogenesis effect in CDAA diet-induced hepatic steatosis

In addition to the assessment of steatosis by NAFLD activity scoring system, using hematoxylin and eosin staining, hepatic steatosis was examined by oil red O staining. A previous report showed that H&E steatosis assessment poorly correlated with triglyceride concentration in the tissue, whereas Oil Red O staining showed much higher sensitivity and specificity for steatosis (23).

**Figure 4:**
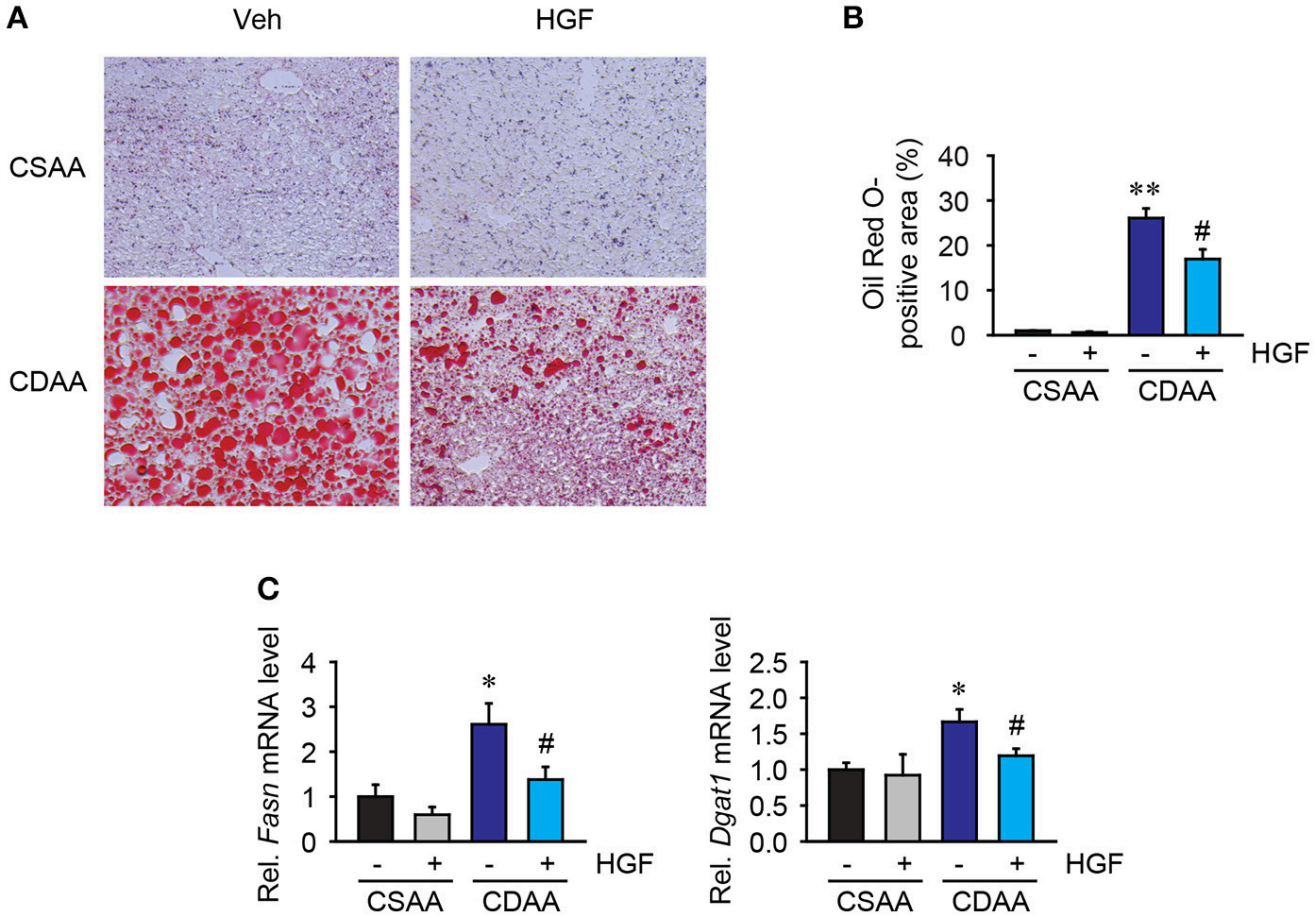
Recombinant HGF protein attenuated CDAA diet-induced lipogenesis. **(A)** Oil red O staining of lipid droplets. Original magnification, x200. **(B)** Quantification of Oil Red O staining. **(C)** Hepatic mRNA expression of *Fasn* and *Dgat1*. Data are presented as mean ± SEM. (*P < 0.05, **P < 0.01, significantly different from CSAA-Veh; ^#^*P* < 0.05, significantly different from CDAA-Veh).

Three weeks of CDAA diet feeding induced accumulation of large lipid droplets in hepatocytes in vehicle-treated mice but, CSAA diet feeding did not (Figures 4A,B). Serum triglyceride and total cholesterol levels were unchanged (Table 2). Seven days of daily administration of recombinant HGF protein significantly suppressed accumulation of lipid droplets in hepatocytes (Figures 4A,B). The study then assessed lipogenesis-related gene expression. Quantitative real-time PCR analysis shows that increased *Fasn* and *Dgat1* genes by CDAA diet feeding were significantly suppressed in mice treated with recombinant HGF protein (Figure 4C). These results suggest that HGF treatment inhibits hepatic lipid accumulation through inhibiting expression of lipogenesis-related genes, such as *Fasn* and *Dgat1*.

**Table 2:** The serum triglyceride and cholesterol profiles.

	**CSAA**		**CDAA**	
	**Vehicle**	**HGF**	**Vehicle**	**HGF**
	**(*n* = 4)**	**(*n* = 3)**	**(*n* = 16)**	**(*n* = 12)**
Triglycerides (mg/dl)	60.3 ± 21.6	68.3 ± 26.8	58.1 ± 14.4	60.2 ± 14.9
Total cholesterol (mg/dl)	94.9 ± 10.0	141.2 ± 45.5	96.8 ± 32.2	95.8 ± 44.0

*Data are presented as mean ± S.D*.

### CDAA diet-induced hepatic inflammation is suppressed by recombinant HGF treatment

**Figure 5:**
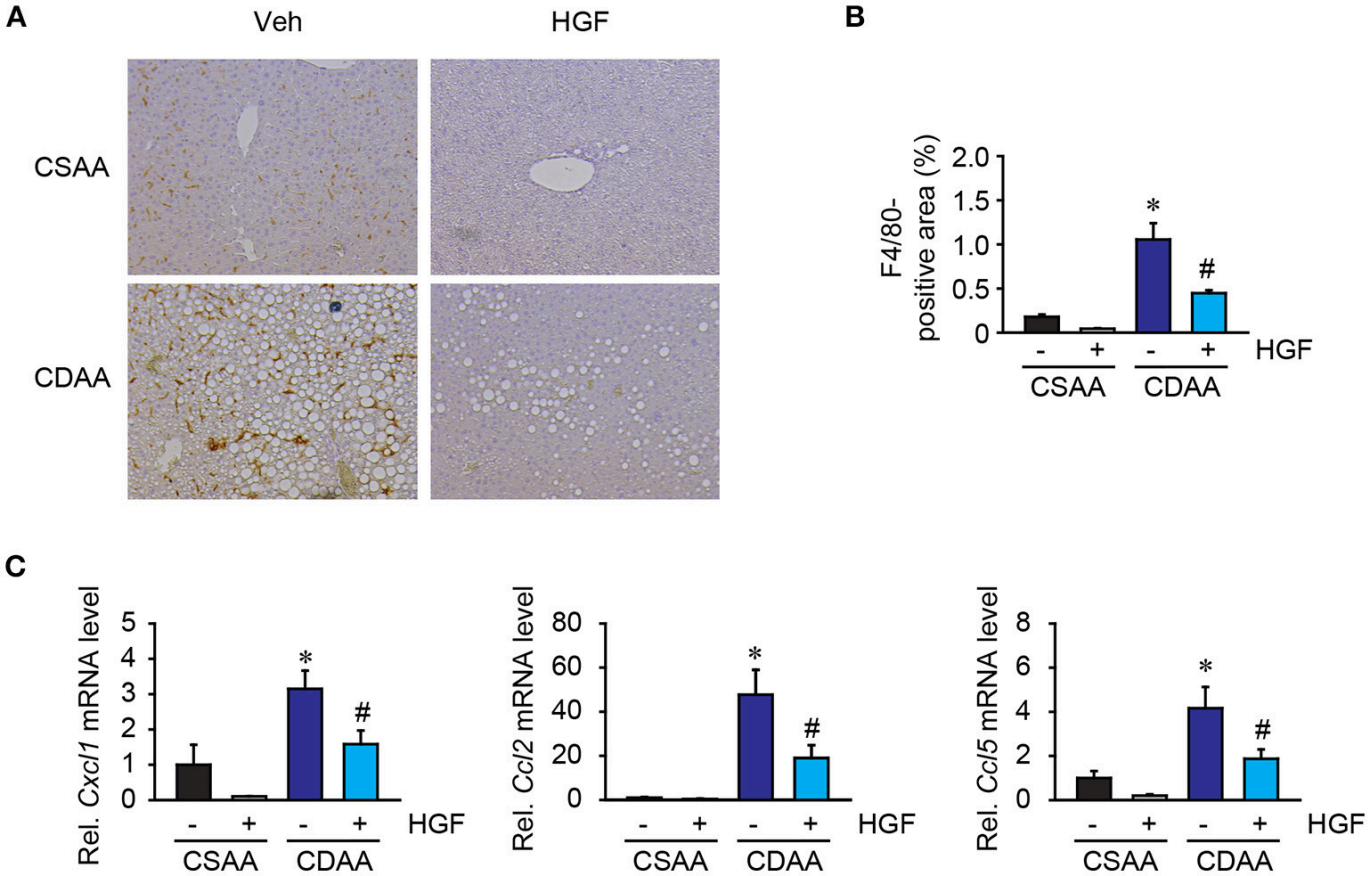
Recombinant HGF protein had anti-inflammatory effect in CDAA diet-induced hepatic steatosis. **(A)** Immunohistochemistry for F4/80. Original magnification, x200. **(B)** Quantification of F4/80 staining. **(C)** Hepatic mRNA expression of *Cxcl1, Ccl2*, and *Ccl5*. Data are presented as mean ± SEM. (*P < 0.05, significantly different from CSAA-Veh; ^#^*P* < 0.05, significantly different from CDAA-Veh).

Inflammatory cell infiltration is a prominent feature of NASH. Among inflammatory cells, hepatic macrophages comprising liver resident Kupffer cells, and bone marrow-derived monocytes are the significant contributor to NASH development. Liver macrophages were examined by immunohistochemical staining for F4/80, a mouse macrophage marker. While basal amounts of liver macrophages were seen in mice fed with CSAA diet, the number of liver macrophages and the foci containing macrophages were significantly augmented in mice fed with 3 weeks of CDAA diet. Interestingly, the recombinant HGF treatment reduced the amounts of liver macrophages in mice fed CDAA diet compared to vehicle treatment (Figures 5A,B). Inflammatory cell infiltration is mainly regulated by the small size (8–10 kDa) of inflammatory cytokines, called chemokines. Increased hepatic levels of *Cxcl1, Ccl2*, and *Ccl5* induced by CDAA diet feeding were significantly reduced by the treatment of recombinant HGF (Figure 5C). Our results suggest that recombinant HGF protein has anti-inflammatory effects on NASH livers that inhibit macrophage infiltration and production of chemokines which play a role in the recruitment of inflammatory cells to injured livers.

### Fibrogenic response induced by CDAA diet was suppressed by recombinant HGF treatment

**Figure 6:**
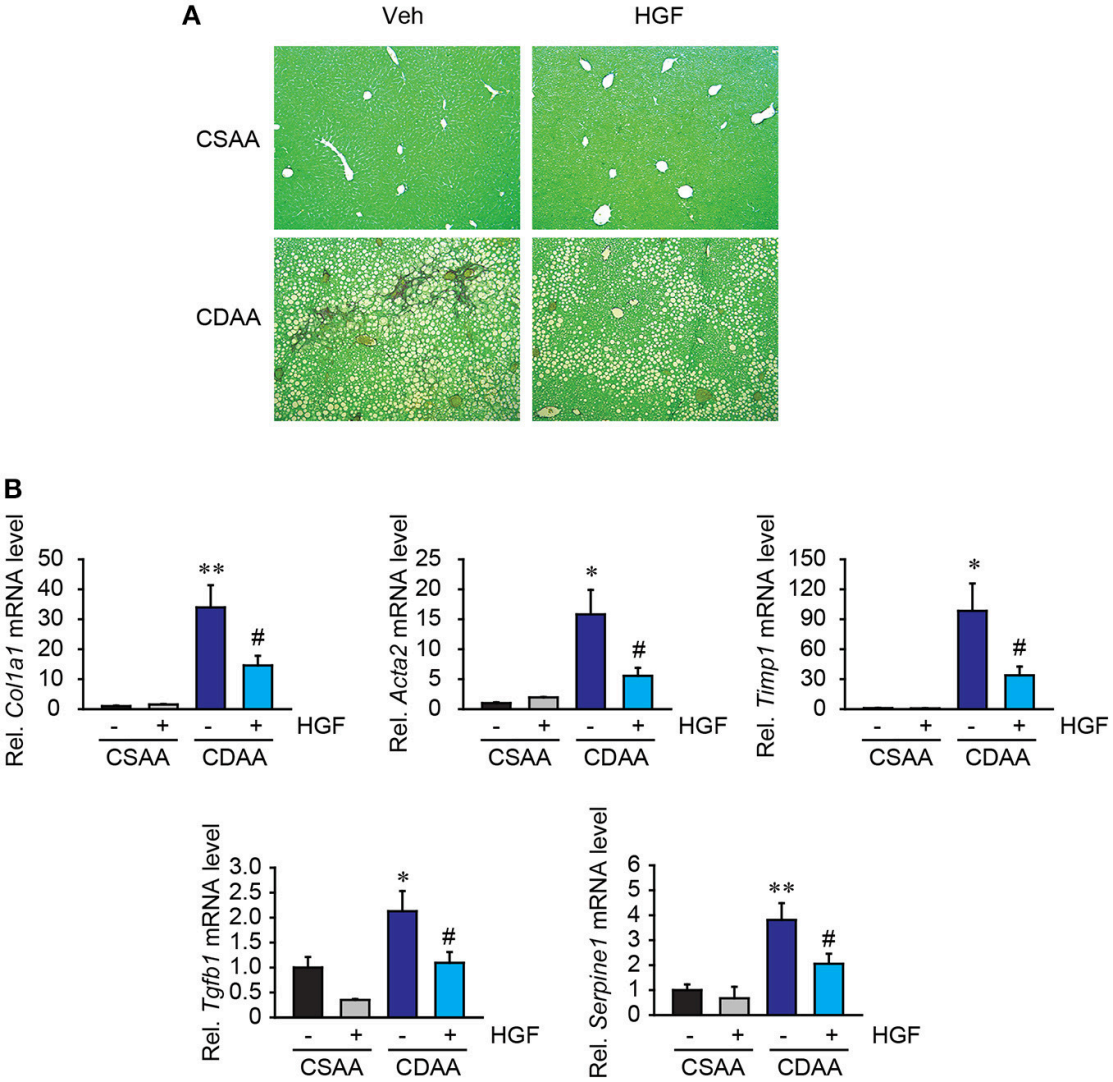
Recombinant HGF ameliorated CDAA diet-induced fibrogenic response. **(A)** Sirius Red staining. Original magnification, x100. **(B)** Hepatic mRNA expression of *Col1a1, Acta2, Timp1, Tgfb1*, and *Serpine1*. Data are presented as mean ± SEM. (*P < 0.05, **P < 0.01, significantly different from CSAA-Veh; ^#^*P* < 0.05, significantly different from CDAA-Veh).

While 3 weeks of CDAA diet feeding does not induce strong fibrillar collagen deposition, very mild collagen expression was seen in liver parenchyma (Figure 6A), which was diminished by recombinant HGF treatment. Notably, there was evidence of upregulation of fibrogenic gene expression in mice with 3 weeks feeding of CDAA. In the vehicle group, CDAA diet feeding significantly upregulated *Col1a1, Acta2, Timp1, Tgfb1*, and *Serpine1* expression in the liver (Figure 6B). On the contrary, in the recombinant HGF treatment group, upregulation of hepatic expression of fibrogenic genes *Col1a1, Acta2, Timp1, Tgfb1*, and *Serpine1* was significantly inhibited (Figure 6B). These results suggest that recombinant HGF treatment can inhibit hepatic stellate cell activation and liver fibrosis progression in NASH.

## Discussion

The present study investigated the therapeutic potential of recombinant HGF protein in the development of NASH in mice. Our data demonstrated that 7 days of intravenous administration of recombinant feline HGF protein is sufficient to suppress the progression of a mouse model of NASH induced by 3 weeks of CDAA diet feeding. The HGF protein treatment significantly suppressed major pathological features of NASH, including the accumulation of lipid droplets in hepatocytes, lipogenesis gene expression, hepatocyte ballooning, elevation of serum ALT levels, inflammatory cell infiltration, chemokine expression, and fibrogenic gene expression. Our study strongly suggests that administration of recombinant HGF protein can be a therapeutic option for NASH.

The physiological role of HGF and its receptor MET in the liver is well-described. HGF is initially produced and released as an inactive precursor form and is normally found in extracellular space. The major producer of HGF in the liver is hepatic stellate cell which is a precursor of myofibroblasts in the liver and contributes to the production of extracellular matrix, progressing to liver fibrosis. During massive liver damage induced by trauma, infection, toxin exposure, and inflammation, pro-HGF is proteolytically cleaved by HGF activator, urokinase-type plasminogen activator, matriptase, and so on, and becomes the biologically active form of HGF (7). The active form of HGF binds to its receptor MET, encoded by proto-oncogene *c-met*, and induces its biological actions, including hepatocyte proliferation, anti-apoptosis, wound healing, and liver tissue homeostasis. Serum HGF levels were elevated in patients with NASH compared to control group (24), proposing a compensatory mechanism responsible for hepatic regeneration. Because of the ability of HGF to induce hepatocyte proliferation and liver regeneration, HGF has been suggested to be a potential therapeutic agent for liver cirrhosis (25).

Hepatocyte damage and liver fibrosis are the key pathological features in NASH. Even though serum HGF levels were increased in NASH patients, it is unclear whether the active form of HGF was also increased in NASH patients. In addition, HGF receptor, MET was underexpressed in human NAFLD livers compared to control livers (26). Our data demonstrated that exogenous HGF treatment suppressed CDAA diet-induced hepatocyte damage and fibrogenic response. Previous studies also demonstrated the protective role of the HGF-MET pathway in liver injury and fibrosis. Exogenous administration of recombinant HGF protein suppresses lipopolysaccharide-induced or Fas-mediated mouse models of fulminant hepatitis (12, 27, 28). In Fas-mediated liver injury model, HGF induces Mcl-1 expression and activation of Akt pathway, resulting in inhibition of hepatocyte apoptosis (28). In addition, HGF stimulates hepatocyte proliferation (Figure 3), which is favorable to the liver repair process. Since one of the major functions of HGF is the induction of matrix metalloproteases (MMPs), such as membrane type 1-MMP and MMP9 to degrade the extracellular matrix, a number of reports demonstrated the therapeutic effect of HGF in liver fibrosis (10, 11, 25, 29). HGF is also known to have an anti-inflammatory property. HGF treatment reduced expression of TNF-α, CCL2, and IL-6 in mouse culture macrophages, in which induction of anti-inflammatory heme oxygenase-1 and IL-10 are partly involved (30, 31). These reports are consistent with our data showing that recombinant HGF treatment inhibited NASH-mediated macrophage infiltration and chemokine expression in the liver.

The protective effect of HGF against lipids in hepatocytes has previously been reported. Recombinant HGF treatment reduced intracellular lipid content, likely by accelerating lipid secretion in hepatocytes through activation of microsomal triglyceride transfer protein and apolipoprotein B (32). Moreover, recombinant HGF treatment inhibited cholesterol overload-mediated hepatocyte lipotoxicity by suppressing production of reactive oxygen species (33). An animal study using hepatocyte-specific *c-met*-deleted mice showed exacerbation of a mouse model of NASH induced by methionine-choline deficient diet (13). All of these reports suggest both exogenous and endogenous HGF-MET system play a critical role in the protection of NAFLD development. Blockade of mineralocorticoid receptor signaling attenuates hepatic steatosis and insulin resistance in a mouse model of obesity. Interestingly, the underlying mechanism of attenuated hepatic steatosis by blocking mineralocorticoid receptor is mediated through the HGF-MET pathway (34). Our data demonstrated that exogenous HGF treatment reduced hepatic steatosis along with reduction of lipogenesis genes, but it appears that the protective mechanism of HGF is not mediated through modulation of lipid degradation pathway as our data did not show any changes in the expression of β-oxidation-related genes (data not shown).

Obesity and fatty liver disease have been often observed in cats and dogs (35, 36). Like in humans, obesity is closely associated with metabolic syndrome. High-fat diet feeding altered the composition of fatty acid in liver tissue and serum (37). The stearic acid-rich high fat diet promoted hepatic lipogenesis in the feline liver (37). Feline hepatic lipidosis, also known as feline fatty liver syndrome, is the most diagnosed liver disease in cats (38). This disease is life-threatening without treatment, but the prognosis would be favored in cases of intensive treatment and adequate therapy. The causes and pathogenic mechanisms are scarcely known. Since our data clearly showed that exogenous HGF treatment efficiently reduced the fat content in the liver and inhibited CDAA diet-induced lipogenesis in mice, further evaluation in large animals may be needed to determine the efficacy and safety of HGF in fatty liver disease.

The MET overexpression and activation play a crucial role in cancer cell proliferation, migration, invasion, and metastasis. This signaling also contributes to drug resistance in tumor microenvironment (39). In HCC, MET is overexpressed (40), but some reports showed HGF is underexpressed (41–45). The effect of HGF on hepatocarcinogenesis is controversial. HGF treatment suppressed HCC cell growth *in vitro* in some reports (46, 47). Exogenous HGF administration or HGF transgenic mouse model showed both pro- and anti-tumorigenic effects of HGF (48–56). Nakanishi et al. tested the long-term effect of exogenous HGF to the NASH mouse model on the occurrence of HCC (56). According to their results, treatment with recombinant human HGF did not increase the overall frequency of HCC. We suggest that NAFLD patients or animals with cancer or individuals with the potential to have cancer are not appropriate for HGF treatment.

Besides the beneficial role of HGF in the liver, HGF is also involved in tissue regeneration, protection, and homeostasis in the kidney and neurons (57). Moreover, HGF is a potent inducer of angiogenesis and has a protective role in the development of cardiovascular diseases through modulation of atherosclerosis (58). Given that the leading cause of death by NASH is cardiovascular diseases (59), targeting of the HGF-MET pathway should have beneficial effects on both liver and heart. Therefore, it is worth considering HGF therapy for NAFLD, but the careful selection of patients or animals may have to be considered due to the capacity of HGF to promote cancer progression.

In summary, the present study demonstrated that exogenous administration of recombinant feline HGF protein has potential to mitigate the development of NASH. Although there is a concern about the role of HGF played in cancer progression, it should be considered for those individuals with low risk of developing HCC (e.g., early stage of NASH fibrosis without cirrhosis). Given that HGF can suppress the risk of cardiovascular disease which is the leading cause of death in NASH patients, exogenous HGF administration may be a favorable option for the treatment of NAFLD.

## Author contributions

YMY, MF, MK, and ES contributed conception and design of the study. YMY, MF, ZW, and FM performed experiments, analysis and interpretation of the data, and statistical analysis. YMY and ES wrote the first draft of the manuscript. All authors contributed to manuscript revision, read and approved the submitted version.

### Conflict of interest statement

The authors have the following interests. The study was supported by Nippon Zenyaku Kogyo Co Ltd, the employer of MF. The remaining authors declare that the research was conducted in the absence of any commercial or financial relationships that could be construed as a potential conflict of interest.

## Abbreviations

ALT: alanine aminotransferase
CDAA: choline-deficient amino acid defined
CSAA: choline-supplemented amino acid defined diet
HCC: hepatocellular carcinoma
H&E: hematoxylin and eosin
HCC: hepatocellular carcinoma
HGF: hepatocyte growth factor
MMP: matrix metalloprotease
NAFLD: non-alcoholic fatty liver disease
NASH: non-alcoholic steatohepatitis
PCNA: proliferating cellular nuclear antigen
PCR: polymerase chain reaction
SMA: smooth muscle actin.

## References

[1] Day CP. Non-alcoholic fatty liver disease: a massive problem. Clin Med (Lond). (2011) 11:176–8. 21526706 10.7861/clinmedicine.11-2-176 PMC5922746

[2] Kneeman JM Misdraji J Corey KE. Secondary causes of nonalcoholic fatty liver disease. Therap Adv Gastroenterol. (2012) 5:199–207. 10.1177/1756283X11430859 22570680 PMC3342568

[3] Sanyal AJ Brunt EM Kleiner DE Kowdley KV Chalasani N Lavine JE. Endpoints and clinical trial design for nonalcoholic steatohepatitis. Hepatology (2011) 54:344–53. 10.1002/hep.24376 21520200 PMC4014460

[4] Tiniakos DG Vos MB Brunt EM. Nonalcoholic fatty liver disease: pathology and pathogenesis. Annu Rev Pathol. (2010) 5:145–71. 10.1146/annurev-pathol-121808-102132 20078219

[5] Chedid MF. Nonalcoholic steatohepatitis: the second leading indication for liver transplantation in the USA. Dig Dis Sci. (2017) 62:2621–2. 10.1007/s10620-017-4724-6 28840385

[6] Webb CB. Hepatic lipidosis: clinical review drawn from collective effort. J Feline Med Surg. (2018) 20:217–27. 10.1177/1098612X18758591 29478399 PMC10816292

[7] Nakamura T Sakai K Nakamura T Matsumoto K. Hepatocyte growth factor twenty years on: much more than a growth factor. J Gastroenterol Hepatol. (2011) 26:188–202. 10.1111/j.1440-1746.2010.06549.x 21199531

[8] Tahara M Matsumoto K Nukiwa T Nakamura T. Hepatocyte growth factor leads to recovery from alcohol-induced fatty liver in rats. J Clin Invest. (1999) 103:313–20. 10.1172/JCI4433 9927491 PMC407897

[9] Sakakura Y Kaibori M Oda M Okumura T Kwon AH Kamiyama Y. Recombinant human hepatocyte growth factor protects the liver against hepatic ischemia and reperfusion injury in rats. J Surg Res. (2000) 92:261–6. 10.1006/jsre.2000.5913 10896832

[10] Kusumoto K Ido A Moriuchi A Katsura T Kim I Takahama Y. Repeated intravenous injection of recombinant human hepatocyte growth factor ameliorates liver cirrhosis but causes albuminuria in rats. Int J Mol Med. (2006) 17:503–9. 16465399

[11] Matsuda Y Matsumoto K Yamada A Ichida T Asakura H Komoriya Y. Preventive and therapeutic effects in rats of hepatocyte growth factor infusion on liver fibrosis/cirrhosis. Hepatology (1997) 26:81–9. 10.1053/jhep.1997.v26.pm0009214455 9214455

[12] Kosai K Matsumoto K Funakoshi H Nakamura T. Hepatocyte growth factor prevents endotoxin-induced lethal hepatic failure in mice. Hepatology (1999) 30:151–9. 10.1002/hep.510300102 10385651

[13] Kroy DC Schumacher F Ramadori P Hatting M Bergheim I Gassler N. Hepatocyte specific deletion of c-Met leads to the development of severe non-alcoholic steatohepatitis in mice. J Hepatol. (2014) 61:883–90. 10.1016/j.jhep.2014.05.019 24845607

[14] Tojima H Kakizaki S Kosone T Horiguchi N Yamazaki Y Sato K. Hepatocyte growth factor overexpression ameliorates liver inflammation and fibrosis in a mouse model of nonalcoholic steatohepatitis. Hepatol Int. (2012) 6:620–30. 10.1007/s12072-011-9301-z 21818687

[15] Kiyama S Yamada T Iwata H Sekino T Matsuo H Yoshida N. Reduction of fibrosis in a rat model of non-alcoholic steatohepatitis cirrhosis by human HGF gene transfection using electroporation. J Gastroenterol Hepatol. (2008) 23:e471–6. 10.1111/j.1440-1746.2007.05111.x 17764530

[16] Yang L Miura K Zhang B Matsushita H Yang YM Liang S. TRIF differentially regulates hepatic steatosis and inflammation/fibrosis in mice. Cell Mol Gastroenterol Hepatol. (2017) 3:469–83. 10.1016/j.jcmgh.2016.12.004 28462384 PMC5403956

[17] Miura K Kodama Y Inokuchi S Schnabl B Aoyama T Ohnishi H. Toll-like receptor 9 promotes steatohepatitis by induction of interleukin-1β in mice. Gastroenterology (2010) 139:323–34.e7. 10.1053/j.gastro.2010.03.052 20347818 PMC4631262

[18] Kruitwagen HS Arends B Spee B Brinkhof B van den Ingh TS Rutten VP. Recombinant hepatocyte growth factor treatment in a canine model of congenital liver hypoplasia. Liver Int. (2011) 31:940–9. 10.1111/j.1478-3231.2011.02513.x 21733083

[19] Kobayashi Y Nakamura N Ishizaka T Masuda K Ohno K Tsujimoto H. Molecular cloning of feline hepatocyte growth factor (HGF) cDNA. J Vet Med Sci. (2001) 63:211–4. 11258464 10.1292/jvms.63.211

[20] Kleiner DE Brunt EM Van Natta M Behling C Contos MJ Cummings OW. Design and validation of a histological scoring system for nonalcoholic fatty liver disease. Hepatology (2005) 41:1313–21. 10.1002/hep.20701 15915461

[21] Song IJ Yang YM Inokuchi-Shimizu S Roh YS Yang L Seki E. The contribution of toll-like receptor signaling to the development of liver fibrosis and cancer in hepatocyte-specific TAK1-deleted mice. Int J Cancer. (2018) 142:81–91. 10.1002/ijc.31029 28875549 PMC5790193

[22] Inokuchi S Aoyama T Miura K Osterreicher CH Kodama Y Miyai K. Disruption of TAK1 in hepatocytes causes hepatic injury, inflammation, fibrosis, and carcinogenesis. Proc Natl Acad Sci USA. (2010) 107:844–9. 10.1073/pnas.0909781107 20080763 PMC2818947

[23] Levene AP Kudo H Armstrong MJ Thursz MR Gedroyc WM Anstee QM. Quantifying hepatic steatosis–more than meets the eye. Histopathology (2012) 60:971–81. 10.1111/j.1365-2559.2012.04193.x 22372668

[24] Agrawal RP Sheroan V Ola V Sulemani AA Singh N Sirohi P. Hepatocyte growth factor, adiponectin and hepatic histopathology in non-alcoholic steatohepatitis. J Assoc Physicians India. (2013) 61:789–92. 24974489

[25] Ueki T Kaneda Y Tsutsui H Nakanishi K Sawa Y Morishita R. Hepatocyte growth factor gene therapy of liver cirrhosis in rats. Nat Med. (1999) 5:226–30. 10.1038/5593 9930873

[26] Zou C Ma J Wang X Guo L Zhu Z Stoops J. Lack of Fas antagonism by Met in human fatty liver disease. Nat Med. (2007) 13:1078–85. 10.1038/nm1625 17704785

[27] Kosai K Matsumoto K Nagata S Tsujimoto Y Nakamura T. Abrogation of Fas-induced fulminant hepatic failure in mice by hepatocyte growth factor. Biochem Biophys Res Commun. (1998) 244:683–90. 9535725 10.1006/bbrc.1998.8293

[28] Schulze-Bergkamen H Brenner D Krueger A Suess D Fas SC Frey CR. Hepatocyte growth factor induces Mcl-1 in primary human hepatocytes and inhibits CD95-mediated apoptosis via Akt. Hepatology (2004) 39:645–54. 10.1002/hep.20138 14999683

[29] Xia JL Dai C Michalopoulos GK Liu Y. Hepatocyte growth factor attenuates liver fibrosis induced by bile duct ligation. Am J Pathol. (2006) 168:1500–12. 10.2353/ajpath.2006.050747 16651617 PMC1606599

[30] Kusunoki H Taniyama Y Otsu R Rakugi H Morishita R. Anti-inflammatory effects of hepatocyte growth factor on the vicious cycle of macrophages and adipocytes. Hypertens Res. (2014) 37:500–6. 10.1038/hr.2014.41 24621470

[31] Kamimoto M Mizuno S Nakamura T. Reciprocal regulation of IL-6 and IL-10 balance by HGF via recruitment of heme oxygenase-1 in macrophages for attenuation of liver injury in a mouse model of endotoxemia. Int J Mol Med. (2009) 24:161–70. 19578789 10.3892/ijmm_00000219

[32] Kosone T Takagi H Horiguchi N Ariyama Y Otsuka T Sohara N. HGF ameliorates a high-fat diet-induced fatty liver. Am J Physiol Gastrointest Liver Physiol. (2007) 293:G204–10. 10.1152/ajpgi.00021.2007 17395903

[33] Dominguez-Perez M Nuno-Lambarri N Clavijo-Cornejo D Luna-Lopez A Souza V Bucio L. Hepatocyte growth factor reduces free cholesterol-mediated lipotoxicity in primary hepatocytes by countering oxidative stress. Oxid Med Cell Longev. (2016) 2016:7960386. 10.1155/2016/7960386 27143995 PMC4842075

[34] Zhang YY Li C Yao GF Du LJ Liu Y Zheng XJ. Deletion of macrophage mineralocorticoid receptor protects hepatic steatosis and insulin resistance through ERalpha/HGF/Met pathway. Diabetes (2017) 66:1535–47. 10.2337/db16-1354 28325853 PMC5860190

[35] German AJ. The growing problem of obesity in dogs and cats. J Nutr. (2006) 136:1940S–6S. 10.1093/jn/136.7.1940S 16772464

[36] Roth L. Comparison of liver cytology and biopsy diagnoses in dogs and cats: 56 cases. Vet Clin Pathol. (2001) 30:35–8. 12024329 10.1111/j.1939-165x.2001.tb00254.x

[37] Fujiwara M Mori N Sato T Tazaki H Ishikawa S Yamamoto I. Changes in fatty acid composition in tissue and serum of obese cats fed a high fat diet. BMC Vet Res. (2015) 11:200. 10.1186/s12917-015-0519-1 26268360 PMC4534048

[38] Kuzi S Segev G Kedar S Yas E Aroch I. Prognostic markers in feline hepatic lipidosis: a retrospective study of 71 cats. Vet Rec. (2017) 181:512. 10.1136/vr.104252 28978714

[39] Imamura R Matsumoto K. Hepatocyte growth factor in physiology and infectious diseases. Cytokine (2017) 98:97–106. 10.1016/j.cyto.2016.12.025 28094206

[40] You H Ding W Dang H Jiang Y Rountree CB. c-Met represents a potential therapeutic target for personalized treatment in hepatocellular carcinoma. Hepatology (2011) 54:879–89. 10.1002/hep.24450 21618573 PMC3181384

[41] Selden C Farnaud S Ding SF Habib N Foster C Hodgson HJ. Expression of hepatocyte growth factor mRNA, and c-met mRNA (hepatocyte growth factor receptor) in human liver tumours. J Hepatol. (1994) 21:227–34. 7989714 10.1016/s0168-8278(05)80400-3

[42] Noguchi O Enomoto N Ikeda T Kobayashi F Marumo F Sato C. Gene expressions of c-met and hepatocyte growth factor in chronic liver disease and hepatocellular carcinoma. J Hepatol. (1996) 24:286–92. 8778194 10.1016/s0168-8278(96)80006-7

[43] Kiss A Wang NJ Xie JP Thorgeirsson SS. Analysis of transforming growth factor (TGF)-alpha/epidermal growth factor receptor, hepatocyte growth Factor/c-met,TGF-beta receptor type II, and p53 expression in human hepatocellular carcinomas. Clin Cancer Res. (1997) 3:1059–66. 9815784

[44] Tavian D De Petro G Benetti A Portolani N Giulini SM Barlati S. u-PA and c-MET mRNA expression is co-ordinately enhanced while hepatocyte growth factor mRNA is down-regulated in human hepatocellular carcinoma. Int J Cancer. (2000) 87:644–9. 10925356

[45] Daveau M Scotte M Francois A Coulouarn C Ros G Tallet Y. Hepatocyte growth factor, transforming growth factor alpha, and their receptors as combined markers of prognosis in hepatocellular carcinoma. Mol Carcinog. (2003) 36:130–41. 10.1002/mc.10103 12619035

[46] Tajima H Matsumoto K Nakamura T. Hepatocyte growth factor has potent anti-proliferative activity in various tumor cell lines. FEBS Lett. (1991) 291:229–32. 1657643 10.1016/0014-5793(91)81291-f

[47] Shiota G Rhoads DB Wang TC Nakamura T Schmidt EV. Hepatocyte growth factor inhibits growth of hepatocellular carcinoma cells. Proc Natl Acad Sci USA. (1992) 89:373–7. 1309612 10.1073/pnas.89.1.373 PMC48239

[48] Liu ML Mars WM Michalopoulos GK. Hepatocyte growth factor inhibits cell proliferation *in vivo* of rat hepatocellular carcinomas induced by diethylnitrosamine. Carcinogenesis (1995) 16:841–3. 7728965 10.1093/carcin/16.4.841

[49] Yaono M Hasegawa R Mizoguchi Y Futakuchi M Nakamura T Ito N. Hepatocyte growth factor enhancement of preneoplastic hepatic foci development in rats treated with diethylnitrosamine and N-ethyl-N-hydroxyethylnitrosamine. Jpn J Cancer Res. (1995) 86:718–23. 7559093 10.1111/j.1349-7006.1995.tb02459.x PMC5920913

[50] Ogasawara H Hiramoto J Takahashi M Shirahama K Furusaka A Hiyane S Hepatocyte growth factor stimulates DNA synthesis in rat preneoplastic hepatocytes but not in liver carcinoma cells. Gastroenterology (1998) 114:775–81. 9516398 10.1016/s0016-5085(98)70591-8

[51] Sakata H Takayama H Sharp R Rubin JS Merlino G LaRochelle WJ. Hepatocyte growth factor/scatter factor overexpression induces growth, abnormal development, and tumor formation in transgenic mouse livers. Cell Growth Differ. (1996) 7:1513–23. 8930401

[52] Horiguchi N Takayama H Toyoda M Otsuka T Fukusato T Merlino G. Hepatocyte growth factor promotes hepatocarcinogenesis through c-Met autocrine activation and enhanced angiogenesis in transgenic mice treated with diethylnitrosamine. Oncogene (2002) 21:1791–9. 10.1038/sj.onc.1205248 11896611

[53] Shiota G Wang TC Nakamura T Schmidt EV. Hepatocyte growth factor in transgenic mice: effects on hepatocyte growth, liver regeneration and gene expression. Hepatology (1994) 19:962–72. 8138271

[54] Santoni-Rugiu E Preisegger KH Kiss A Audolfsson T Shiota G Schmidt EV. Inhibition of neoplastic development in the liver by hepatocyte growth factor in a transgenic mouse model. Proc Natl Acad Sci USA. (1996) 93:9577–82. 8790372 10.1073/pnas.93.18.9577 PMC38470

[55] Goyal L Muzumdar MD Zhu AX. Targeting the HGF/c-MET pathway in hepatocellular carcinoma. Clin Cancer Res. (2013) 19:2310–8. 10.1158/1078-0432.CCR-12-2791 23388504 PMC4583193

[56] Nakanishi C Moriuchi A Ido A Numata M Kim ID Kusumoto K. Effect of hepatocyte growth factor on endogenous hepatocarcinogenesis in rats fed a choline-deficient L-amino acid-defined diet. Oncol Rep. (2006) 16:25–31. 16786119

[57] Matsumoto K Funakoshi H Takahashi H Sakai K. HGF-Met pathway in regeneration and drug discovery. Biomedicines (2014) 2:275–300. 10.3390/biomedicines2040275 28548072 PMC5344275

[58] Madonna R Cevik C Nasser M De Caterina R. Hepatocyte growth factor: molecular biomarker and player in cardioprotection and cardiovascular regeneration. Thromb Haemost. (2012) 107:656–61. 10.1160/TH11-10-0711 22318499

[59] Adams LA Anstee QM. A fatty liver leads to a broken heart? J Hepatol. (2016) 65:14–6. 10.1016/j.jhep.2016.03.012 27025686

